# Serum peptidome profiles immune response of COVID-19 Vaccine administration

**DOI:** 10.3389/fimmu.2022.956369

**Published:** 2022-08-24

**Authors:** Wenjia Zhang, Dandan Li, Bin Xu, Lanlan Xu, Qian Lyu, Xiangyi Liu, Zhijie Li, Jian Zhang, Wei Sun, Qingwei Ma, Liang Qiao, Pu Liao

**Affiliations:** ^1^ Department of Clinical Laboratory, Chongqing General Hospital, Chongqing, China; ^2^ Department of Chemistry, Fudan University, Shanghai, China; ^3^ Bioyong Technologics, Inc., Beijing, China; ^4^ Department of Laboratory Medicine, Beijing Tongren Hospital, Capital Medical University, Beijing, China; ^5^ State Key Laboratory of Proteomics, Beijing Proteome Research Center, National Center for Protein Sciences (Beijing), Beijing Institute of Lifeomics, Beijing, China

**Keywords:** COVID-19, vaccine, MALDI-TOF, peptidome, serum, immune response

## Abstract

**Background:**

Coronavirus disease 2019 (COVID-19) caused by severe acute respiratory syndrome coronavirus 2 (SARS-CoV-2) has caused significant loss of life and property. In response to the serious pandemic, recently developed vaccines against SARS-CoV-2 have been administrated to the public. Nevertheless, the research on human immunization response against COVID-19 vaccines is insufficient. Although much information associated with vaccine efficacy, safety and immunogenicity has been reported by pharmaceutical companies based on laboratory studies and clinical trials, vaccine evaluation needs to be extended further to better understand the effect of COVID-19 vaccines on human beings.

**Methods:**

We performed a comparative peptidome analysis on serum samples from 95 participants collected at four time points before and after receiving CoronaVac. The collected serum samples were analyzed by matrix-assisted laser desorption/ionization time-of-flight mass spectrometry (MALDI-TOF MS) to profile the serum peptides, and also subjected to humoral and cellular immune response analyses to obtain typical immunogenicity information.

**Results:**

Significant difference in serum peptidome profiles by MALDI-TOF MS was observed after vaccination. By supervised statistical analysis, a total of 13 serum MALDI-TOF MS feature peaks were obtained on day 28 and day 42 of vaccination. The feature peaks were identified as component C1q receptor, CD59 glycoprotein, mannose-binding protein C, platelet basic protein, CD99 antigen, Leucine-rich alpha-2-glycoprotein, integral membrane protein 2B, platelet factor 4 and hemoglobin subunits. Combining with immunogenicity analysis, the study provided evidence for the humoral and cellular immune responses activated by CoronaVac. Furthermore, we found that it is possible to distinguish neutralizing antibody (NAbs)-positive from NAbs-negative individuals after complete vaccination using the serum peptidome profiles by MALDI-TOF MS together with machine learning methods, including random forest (RF), partial least squares-discriminant analysis (PLS-DA), linear support vector machine (SVM) and logistic regression (LR).

**Conclusions:**

The study shows the promise of MALDI-TOF MS-based serum peptidome analysis for the assessment of immune responses activated by COVID-19 vaccination, and discovered a panel of serum peptides biomarkers for COVID-19 vaccination and for NAbs generation. The method developed in this study can help not only in the development of new vaccines, but also in the post-marketing evaluation of developed vaccines.

## Introduction

Coronavirus disease 2019 (COVID-19) has caused considerable loss of life and property since its outbreak. To curb the pandemic, physical strategies, such as personal protective equipment distribution, social distancing rules and quarantine policies, have been widely implemented. Compared with physical strategies, herd immunity is a critical approach to control the pandemic. To date, different types of COVID-19 vaccines have been developed, approved, and widely distributed across the world, such as mRNA vaccines (Pfizer–BioNTech and Moderna), adenovirus vector vaccines (Oxford–AstraZeneca), inactivated virus vaccines (Sinovac and Sinopharm), etc. (1). Based on these licensed vaccine products, herd immunization has been boosted. As of July 2022, University of Oxford confirmed that more than 12 billion doses of COVID-19 vaccine have been administered worldwide (2) and the number is increasing continuously. Nevertheless, in response to the urgent and huge demand for COVID-19 vaccines, the development cycle of vaccines was greatly compressed (3). Besides, the natural immune response induced by severe acute respiratory syndrome coronavirus 2 (SARS-CoV-2) is still not fully understood. Given the paucity of development experience and the greatly shortened vaccine development time, there is a lack of thorough knowledge on the immune response induced by COVID-19 vaccination.

Vaccine efficacy and safety are the most important factors to be considered (4, 5). The immunogenicity of vaccine is frequently evaluated, involving both humoral and cellular immune response analyses (6). Immunogenicity is a sophisticated and informative indicator that is associated with the vaccine-induced immune responses and their change over time (6). For the assessment of immunogenicity, production of antibody is measured (7). Antibody monitoring, particularly on total antibody (TAbs) and neutralizing antibody (NAbs), is the primary laboratory strategy to test the protective ability of vaccines against SARS-CoV-2 (8, 9). There are several methods to measure NAbs. The common ones are plaque reduction neutralization test (PRNT), fluorescent neutralization assay (FNA), microneutralization assay (MNA), pseudovirus neutralization assay (PSVNA), and surrogate virus neutralization test (SVNT) (10). PRNT is the gold standard to evaluate immune protection using live virus (11), but need to be accomplished in bio-safety level 3 (BSL-3) laboratory, which limits its application scenario (8). FNA can provide equivalent results to PRNT, but sometimes it also needs to be performed in BSL-3 laboratory (8). MNA involves live SARS-CoV-2 virus as well, and is limited not only by safety risks but also by the disadvantages of time-consuming and high cost. Compared to PRNT, MNA and FNA, PSVNA is a safe method to test NAbs in Biosafety Level 2 (BSL2) laboratory by using pseudo virus without replicating ability. PSVNA is of high sensitivity, accuracy, and repeatability (12), but requires complicated procedure. SVNT has the characteristics of high-throughput and easy-to-operate without the requirement of live virus, and it also detects designated NAbs (8). To date, the currently used methods of NAbs analysis are mainly limited by the requirement of bio-safety laboratory, throughput, time costs and economic costs (8, 11, 13). Development of new strategies is of interest for the ongoing vaccine evaluation work with mass vaccination efforts underway.

Apart from antibody monitoring, cellular immune responses are also widely monitored for vaccine development (14, 15). Prendecki et al. showed that mRNA (BNT162b2, mRNA-1273) and viral-vector vaccines can elicit strong T cell response against SARS-CoV-2 (16). A recent article evaluated the T cell response induced by BBIBP-CorV (Sinopharm, inactivated virus) with a focus on IFN-γ (17). Recently, Zhao et al. reported the humoral and cellular immune responses of 136 participants activated by two-dose of CoronaVac after 1, 3, 6, and 12 moths (18). Jiang et al. reported the immune features of CoronaVac based on 13 healthy people and 12 people that recovered from COVID-19 infection (19). Although pharmaceutical companies have reported the efficacy, safety and immunogenicity of COVID-19 vaccines based on laboratory studies and clinical trials, additional vaccine evaluation is needed to better understand the effect of COVID-19 vaccines on human beings, and new strategies for the evaluation of immune response against vaccines are needed.

Matrix-assisted laser desorption/ionization time-of-flight mass spectrometry (MALDI-TOF MS) is widely used in clinical scenarios, particularly microbial identification and biomarker discovery, due to its advantages, such as rapid analysis, high-throughput, easy operation, low-cost in consumables, etc. The peptide mass fingerprint generated by MALDI-TOF MS can describe a complex mixture of proteolytically derived peptides in human body fluids associated with biological events happening throughout the whole body. Many studies have reported that MALDI-TOF MS can be used to diagnose diseases, such as multiple myeloma (20), liver cancer (21), prostate cancer (22), active mycobacterium tuberculosis (Mtb) infection (23), etc., by screening a panel of protein and peptide biomarkers in human body fluids. Responding to the COVID-19 pandemic, MALDI-TOF-based serum peptides fingerprint, i.e., MALDI-TOF-based serum peptidome profiling, has been developed for the rapid screening of COVID-19 infectious people and COVID-19 illness severity (24, 25). MALDI-TOF MS combining liquid chromatography-tandem mass spectrometry (LC-MS/MS) has also been performed on serum and nasopharyngeal swabs from COVID-19 patients (24–27) to identify potential biomarkers of SARS-CoV-2 infection. Herein, we performed a comparative peptidome analysis on human serum before and after receiving CoronaVac.

## Materials and methods

### Sample collection and storage

Ninety-five participants were recruited. The subjects had no previous infection with COVID-19 and no COVID-19 vaccination before the study. The whole sampling was accomplished between February and April 2021. During this period, all participants were injected with a two-dose COVID-19 inactivated vaccine (CoronaVac, Sinovac Biotech Co., Ltd, Beijing, China) in Chongqing General Hospital, and four batches of blood samples were collected before and after vaccination. The sampling time was determined as day 0 (on the day but before the first injection), day 21 (3 weeks after the first injection, and on the day but before the second injection), day 28 (1 week after the second injection) and day 42 (3 weeks after the second injection). After centrifugation at 2264 g for 10 min and sterilization at 56°C for 30 min, the serum samples were aliquoted and frozen at −80°C until use. Erythrocyte-lysed whole blood was prepared from the samples collected on day 0 and day 28, and immediately subjected to lymphocytes subpopulation analysis.

### Immunogenicity profiling

TAbs and NAbs were analyzed for all the collected serum at the four time points. TAbs were quantified using TAbs chemiluminescence reagent kits (Xiamen Wantaicare Company, batch 20210101, Xiamen, China) on an automated chemiluminescence immunoassay analyzer (Xiamen Youmic Company, Caris 200, Xiamen, China). Negative and positive TAbs were determined according to the instructions provided with the kit. NAbs were quantified using chemiluminescence reagent kits targeting at SARS-CoV-2 receptor binding domain (RBD) (Shenzhen Yahuilong company reagent, batch 20210101, Shenzhen, China) on an automated chemiluminescence analyzer (Shenzhen Yahuilong Biotechnology Co., Ltd., iFlash 3000-A, Shenzhen, China). Negative and positive NAbs were determined according to the instructions provided with the kit.

Cytokines were analyzed for serum sample collected on day 0 and day 28 by cytokines assay kits (Weimi Bio-Tech Co., Guangzhou, China) using a BD FACS CantoII flow cytometer (Becton, Dickinson and Company, Franklin Lakes, New Jersey, U.S.) following the manufacturer’s’ instructions. The kits included 14 types of microbeads with distinct fluorescence intensities and coated with, respectively, specific antibodies against IL-17F, IL-21, IL-2, IL-4, IL-5, IL-6, IL-8, IL-1β, IL-17A, IL-10, TNF-α, TNF-β, IL-12p70 and IFN-γ. After incubation with serum sample, the immunocomplex was further combined with PE fluorescently labeled detection antibody to form a double-antibody sandwich complex, and the fluorescence intensity of the complex was analyzed by flow cytometer to quantify the cytokines. The data were analyzed using the FCAP Array Software v3.0.

Lymphocytes subpopulation were analyzed by a MultiTEST TM IMK kit (Becton, Dickinson and Company, Franklin Lakes, New Jersey, U.S.) using a BD FACS CantoII flow cytometer (Becton, Dickinson and Company, Franklin Lakes, New Jersey, U.S.) following the manufacturer’s’ instructions. Absolute counts of T lymphocytes (CD3+), B lymphocytes (CD19+), helper T lymphocytes (CD3+CD4+), cytotoxic T lymphocytes (CD3+CD8+), and natural killer (NK) cells (CD16+CD56+) were quantified in erythrocyte-lysed whole blood samples collected on day 0 and day 28.

### MALDI-TOF MS analysis

For all the samples, 5 µl serum was diluted 10 times using dilution buffer (PMFpre kit 1010305, Bioyong Technologies Inc., Beijing, China), and 10 µl of the diluted serum was mixed with 10 µl sinapinic acid matrix. One µl of the mixture was dropped on a sample spot of a stainless-steel target plate. The sample was dried at room temperature followed by MALDI-TOF MS (Clin-TOF-II; Bioyong Technologies Inc., Beijing, China) analysis under the linear positive mode. The mass spectrometer was calibrated with a standard calibration mixture of peptides and proteins. The calibration tolerance was 500 ppm. The mass range was m/z 3,000 to m/z 30,000. Each spectrum was accumulated from 50 positions of a sample spot with 10 laser shots per position.

### MALDI-TOF MS data processing and analysis

Raw data of MALDI-TOF MS were processed by an R package, MALDIquant (28), with operations including sqrt transformation, savitzkyGolay smoothing with a halfWindowsize of 5, and SNIP baseline correction. Then, peak detection was performed using the MAD method with a halfWindowsize of 20 and a signal-to-noise (S/N) threshold of 6. Minifrequency was set as 0.25. Peaks were then binned by binPeaks with a tolerance of 0.005 for all samples. Mass range was from 3,000 m/z to 30,000 m/z. The data obtained as a matrix table was then further processed by log 2 transformation, quantile normalization, and missing values imputing using Metaboanalyst (29) (McGill University, Montreal, Canada, https://www.metaboanalyst.ca/).

### Statistical analysis

Unsupervised and supervised statistical analysis was performed using Metaboanalyst, including principal component analysis (PCA), partial least squares-discriminant analysis (PLS-DA) and volcano plot. Significant MALDI-TOF feature peaks were determined by PLS-DA (VIP > 2.0) and volcano plot (FC > 1.5, p-value < 0.05). P-value was calculated by the Wilcoxon test to confirm the significant difference of feature peaks between the same set of people at different time points, or by the Mann-Whitney test to confirm the significant difference of feature peaks between two independent sets of samples, i.e., between the NAbs positive and negative groups.

### Proteomic analysis

The serum samples from three participants (Sample 23, 41, 78) were selected for proteomic analysis, since the samples showed intensive significant MALDI-TOF MS feature peaks (identified using the statistical analysis method described in 2.5). The samples were mixed, and four mixed samples at four collection time points were obtained (day 0, day 21, day 28 and day 42). For each sample, abundant proteins were removed using High Select Top14 Abundant Protein Depletion Mini Spin Columns (A36370, Thermo Fisher Scientific, Waltham, MA, USA). The remaining samples were then dissolved in a protein lysis solution containing 8M urea (U6504, Sigma-Aldrich Co., St. Louis, MO, USA) and 0.1% SDS (L6026, Sigma-Aldrich Co., St. Louis, MO, USA). BCA quantification kit (P0010, Beyotime Biotechnology, Beijing, China) was used to quantify the final protein concentration of all samples. After tris (2-carboxyethyl) phosphine (TCEP, Thermo Fisher Scientific, Waltham, MA, USA) reduction at 37°C for 1 h and iodoacetamide (IAA, Sigma-Aldrich Co., St. Louis, MO, USA) alkylation at 25°C for 1 h, 6 volume of pre-cooled acetone was added to precipitate proteins at −20°C for 4 h. The precipitate was collected by centrifugation and washed twice with 90% pre-cooled acetone. Then the protein samples were re-dissolved in 100 μl ammonium bicarbonate solution (25 mM). Proteome sequencing grade trypsin (Hualishi scientific, Beijing, China) was added at 1:50 (w:w) enzyme-to-sample ratio for protein digestion at 37°C overnight. After digestion, a C18 column (Thermo Fisher Scientific, Waltham, MA, USA) was used for desalting followed by peptide quantification using Pierce™ quantitative colorimetric peptide assay (Thermo Fisher Scientific, Waltham, MA, USA). Afterwards, the samples were lyophilized by an LNG-T98 freeze concentration centrifugation dryer (Taicang Huamei, Taicang, Jiangsu, China) for further LC-MS/MS analysis.

The processed samples were then redissolved in 0.1% formic acid (FA) aqueous solution (solvent A) to a concentration of 0.5 μg/μl. Four μL of the sample was injected into an Orbitrap Fusion Lumos spectrometer (Thermo Fisher Scientific, Waltham, MA, USA) coupled with a nanoLC system (Thermo-ESAY-nLC, Thermo Fisher Scientific, Waltham, MA, USA) using a 25 cm analytical column (75 μm inner diameter, 1.9 μm resin, Dr Maisch, Ammerbuch-Entringen, Germany), and separated using a 120-minute gradient. The flow rate of the nanoLC was maintained at 600 nL/min and the column temperature was 50°C. Water and ACN (both containing 0.1% FA) were used as solvents A and B, respectively, with the following gradient elution program: 0-4-79-108-110-120 min, 4%-7%-20%-30%-90%-90% of solvent B, with an electrospray voltage of 2.2 kV. The mass spectrometer was operated in data independent acquisition mode with MS and MS/MS automatically switched. The parameters were (1) MS: scan range (m/z) = 350-1500; resolution = 120,000; AGC target = 40,0000; maximum injection time = 50 ms; (2) HCD-MS/MS: resolution = 30,000; AGC target = 20,000; collision energy = 32%; maximum injection time = 72 ms (3) DIA: Variable isolation windows; 1 m/z overlap per window; number of windows = 60.

Raw MS data were used to search against the Homo sapiens database downloaded from UniProtKB (20,600 entries) using Spectronaut (version 15.2, Biognosys, Schlieren, Switzerland). Default parameters were retained except the quantitative method was set as the first level.

### Annotation of MALDI-TOF MS feature peaks and bioinformatic analysis

Matching between the MALDI-TOF mass spectra feature peaks and the proteomic analysis results was performed under the criteria: the molecular weight of an identified protein/protein fragment was consistent with the m/z of the MALDI-TOF MS peak within a tolerance of 2000 ppm; only the charge state of 1+ was considered for MALDI-TOF MS peaks; the quantity changes of the protein/protein fragment by LC-MS/MS showed the same trend as the intensity change of the matched MALDI-TOF MS peak. If more than one protein/protein fragment matched to the feature peak under the given criteria, priority was given to the protein fragments containing N or C terminal, and then the one with the smallest mass difference. The gene ontology (GO) enrichment analysis was performed by Metascape (30) involving all the identified features.

## Results

### Cohort establishment and immunogenicity analysis

**Figure 1:**
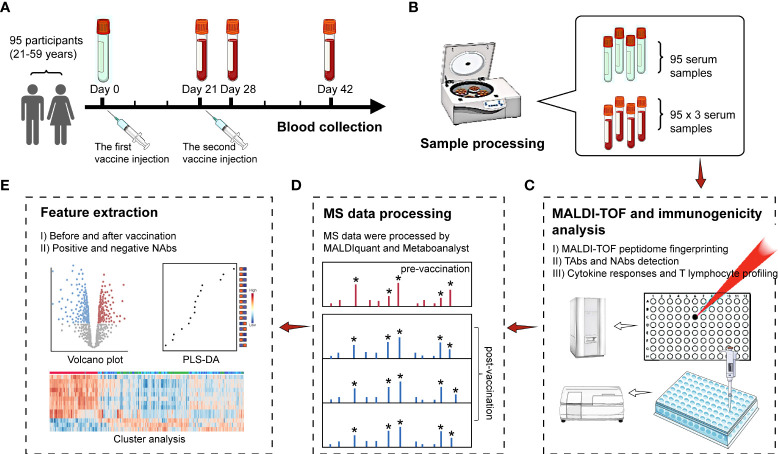
Study design. **(A)** Ninety-five participants were recruited and received two doses of CoronaVac vaccines. Blood samples were collected at four time points before and after vaccine injection. **(B)** Serum separation. **(C)** MALDI-TOF MS and immunogenicity analysis on all the collected serum samples. **(D)** MALDI-TOF MS data processing using MALDIquant and Metaboanalyst. **(E)** Feature selection based on volcano plot and variable importance in projection (VIP) scores by PLS-DA. Hierarchical cluster analyses (HCA) showed the intensity distribution of the selected features among samples. Peaks with an asterisk are peaks of higher intensity filtered by the signal-to-noise ratio and are included in the analysis. Peaks without an asterisk are by default noise peaks and are not included in the analysis.

An overview of the study design is shown in **Figure 1**. Ninety-five participants (21-59 years) accepted two doses of CoronaVac vaccines. Among the 95 participants, the percentage of male (42%) and female (58%) was balanced, with 29% people aged 20-30, 17% people aged 30-40, 19% people aged 40-50 and 35% aged over 50 years (**Table S1**). Regarding past medical history, one of the subjects had coronary artery disease and four had hypertension or diabetes. After vaccination, records showed few symptoms of adverse reactions among the 95 participants. There were two cases of muscle aches, three cases of injection site pain or itching, one case of dizziness and palpitations, without any fever cases. More demographic information of the participants can be found in **Table S1**. Blood samples were collected from the participants during the 6-week recovery phase. Pre-vaccinated blood samples were also collected as the control group. Finally, four batches of blood samples were collected on day 0 (on the day but before the 1st injection), day 21 (on the day but before the 2nd injection), day 28 and day 42 (**Figure 1A**). Sera were separated from the blood samples (**Figure 1B**) and analyzed by MALDI-TOF MS (**Figure 1C**). Statistical analysis was performed on the MALDI-TOF mass spectra for the identification of significant features related to CoronaVac vaccination and NAbs generation (**Figures 1D, E**).

The immunogenicity profiling of antibodies, cytokines and lymphocyte was performed to quantify TAbs, NAbs, IL-17F, IL-21, IL-2, IL-4, IL-5, IL-6, IL-8, IL-1β, IL-17A, IL-10, TNF-α, TNF-β, IL-12p70, IFN-γ, CD3+, CD19+, CD3+CD4+, CD3+CD8+, and CD16+CD56+ (**Figure 1C**). From **Figure S1A**, it can be observed that the positive rates of TAbs (TAbs positive: s/co ≥ 1.00) and NAbs (NAbs positive: ≥10.00 AU/mL) were zero on day 0 and were both below 40% on day 21. TAbs positive rate climbed up to over 80% on day 28 and over 90% on day 42. The high positive rate of TAbs on day 42 indicated that the immune response was activated by COVID-19 vaccination for most participants. For NAbs, the positive rate was consistently below 50% until day 28, and ascended to 73% on day 42, with 26 people still NAbs negative. Within the 26 negative NAbs people, the distribution of female (54%) and male (46%) was almost even (**Figure S1B**). However, more than half (54%) of the people were over 50 years old (**Figure S1C**), while the people > 50 years old only accounted 35% of the whole cohort. People aged 50-59 had lower rates of NAbs production than those aged under 50 (**Table S1**).

Serum cytokine profiling revealed that IL-21 and IL-8 increased significantly comparing day 28 to day 0, while IL-17F, IL-17A, IL-10, IL-12p70, TNF-α, TNF-β and IFN-γ decreased significantly, when considering all the participants (**Figure S2**). When considering separately the group with age > 50 and those with age < 50, the overall regulation trend kept consistent but IL-21, IL-17A, IL-10 and IFN-γ were only significant in the group with age < 50 (**Figures S3** and **S4**). Lymphocyte subpopulation analysis demonstrated the significant up-regulation of CD3+CD4+ and significant down-regulation of CD3+CD8+, CD19+ and CD16+CD56+ comparing day 28 to day 0 considering all the participants (**Figure S2**). The significant regulation of CD19+ and CD16+CD56+ were only observed for the group with age < 50; while the significant regulation of CD3+CD4+ was only observed for the group with age > 50 (Figre S3 and S4).

### Vaccination-induced serum peptidome change

**Figure 2:**
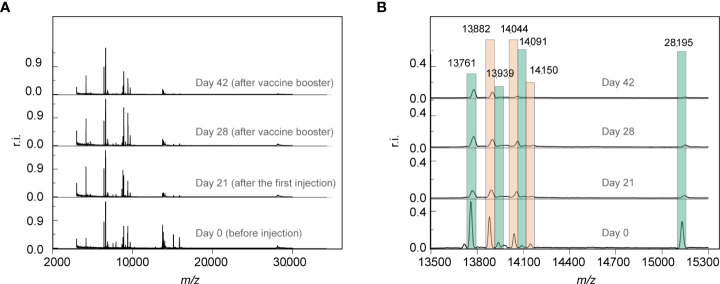
**(A)** The representative MALDI-TOF mass spectra of one participant at four time points pre-vaccination and post-vaccination. **(B)** Partial enlarged view of **(A)**. The mass spectra were normalized against the strongest peak. r.i.: relative intensity.

A total of 380 mass spectra were collected from the 95 participants. The extracted peaks of the mass spectra are shown in supplementary Data S1. After vaccination, the number of peaks increased (**Figure S5**), indicating the increase of serum peptides expression. 327 peaks were obtained after peak alignment within the mass range of m/z 3,000 to m/z 30,000 (m/z, mass-to-charge ratio). Raw mass spectra of Sample 58 at four time points are shown in **Figure 2**. A global view of the four MALDI-TOF mass spectra revealed a high similarity of serum peptidome pattern before and after vaccination (**Figure 2A**). Nevertheless, a few peaks, such as m/z 13,761, m/z 13,882, m/z 13,939, m/z 14,044, m/z 14,091, m/z 14,150 and m/z 28,195 were downregulated after vaccination (**Figure 2B**).

**Figure 3:**
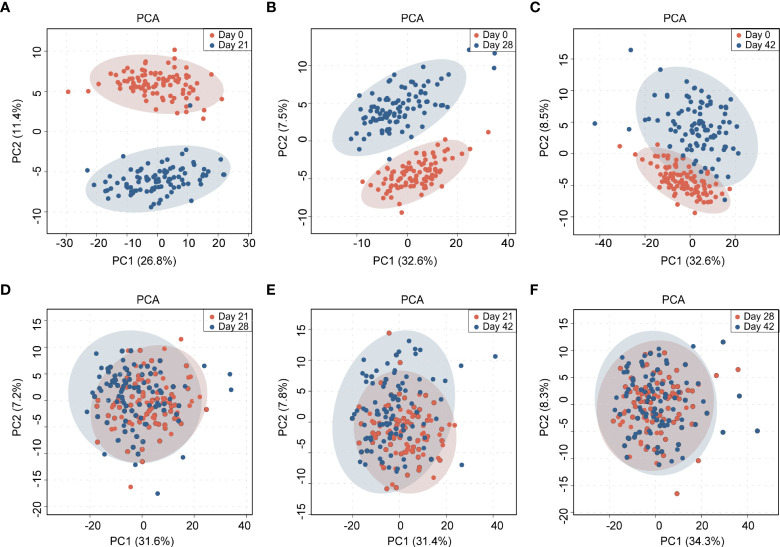
Principal component analysis (PCA) of the MALDI-TOF MS-based serum peptidome profiles collected at 4 time points before and after vaccination: **(A)** between day 0 and day 21; **(B)** between day 0 and day 28; **(C)** between day 0 and day 42; **(D)** between day 21 and day 28; **(E)** between day 21 and day 42; **(F)** between day 28 and day 42. The shadow ovals represent 95% confidence interval.

Principal component analysis (PCA) was performed on the collected serum peptidome profiles. As shown in **Figure 3**, day 0 can be clearly discriminated from day 21, day 28 and day 42. The degree of discrimination diminished with the increase of time interval. Day 0 and day 21 presented the largest difference. In contrast, the MALDI-TOF mass spectra from day 21, day 28 and day 42 cannot be well distinguished by PCA (**Figure 3**). These results demonstrated that the serum peptidome was significantly changed before and after vaccination, the changes were more significant in short term, and the differences were not from batch bias effect. To further demonstrate the classification, a heat map of cluster analysis was performed on the collected serum peptidome profiles, which showed that the pre-vaccine samples clustered mostly together (**Figure S6**).

**Figure 4:**
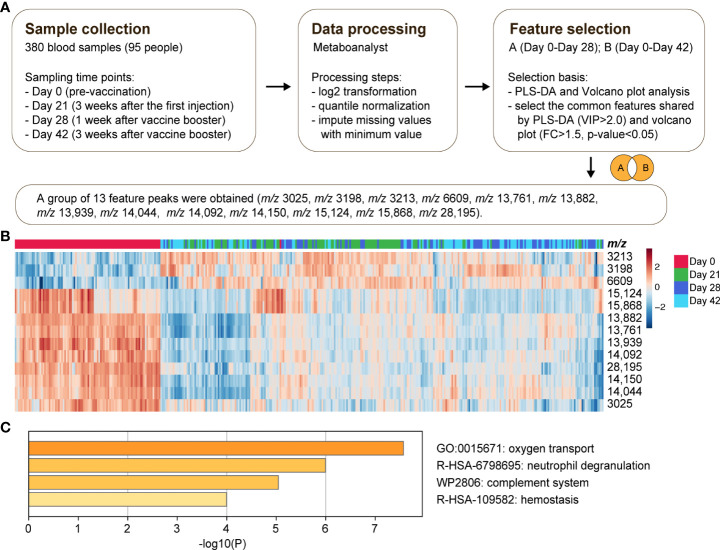
Selection of feature peaks of vaccination. **(A)** A general scheme of sample collection, data processing and feature selection. **(B)** Cluster analysis of the 13 feature peaks of vaccination among all the samples. **(C)** The Gene Ontology (GO) enrichment analysis by Metascape involving all the identified features.

With the significant difference in serum peptidome before and after vaccination, significant MALDI-TOF MS features associated with the vaccination were mined. The feature selection procedure is illustrated in **Figure 4A**. Day 28 and day 42 with full doses of vaccination were chosen to identify distinctive features compared to day 0. The top 15 contributory features with the highest VIP scores in PLS-DA analysis between day 0 and day 28 are shown in **Figure S7A**. The up and down regulations of mass spectrometry features by volcano-plot analysis between day 0 and day 28 are shown in **Figure S7B**. By taking the intersection of the candidate features selected by both PLS-DA and volcano-plot, a group of 12 candidate significant features between day 0 and day 28 was obtained (**Table S2**). Similarly, the same inclusion criteria were used for feature selection between day 0 and day 42 (**Figure S7C** for PLS-DA and **Figure S7D** for volcano-plot analysis). A group of 11 candidate features was obtained (**Table S3**). Combining the features between day 0 and day 28, as well as between day 0 and day 42, a panel of 13 vaccine features were determined. Cluster analysis of the 13 feature peaks among all the samples is visualized as a heat map (**Figure 4B**). It was observed that most of the 13 feature peaks were downregulated after vaccine injection. The downregulated peaks include m/z 3025, m/z 13,761, m/z 13,882, m/z 13,939, m/z 14,044, m/z 14,092, m/z 14,150, m/z 15,124, m/z 15,868 and m/z 28,195. Only three peaks were upregulated, including m/z 3198, m/z 3213 and m/z 6609. The relative intensities of the 13 feature peaks before vaccination and during the 6-week recovery phase after vaccination are shown in **Figure S8**. Proteomic analysis was performed to identify the 13 MALDI-TOF MS feature peaks of vaccination. The features were identified as component C1q receptor (m/z 6609), CD59 glycoprotein (m/z 14,150), mannose-binding protein C (MBL) (m/z 14,092), platelet basic protein (m/z 13,882), CD99 antigen (m/z 3025), Leucine-rich alpha-2-glycoprotein (LRG1) (m/z 28,195), and hemoglobin (Hb) subunits (m/z 14,044, m/z 15,124 and m/z 15,868) (**Table S4**). The identified proteins showed significant down-regulation after vaccination, except component C1q receptor. Gene ontology (GO) enrichment analysis revealed that these identified proteins have a close relationship with the pathways of oxygen transport, neutrophil degranulation, complement system, and hemostasis (**Figure 4C**).

### Correlation between serum peptidome profile and NAbs generation by vaccination

**Figure 5:**
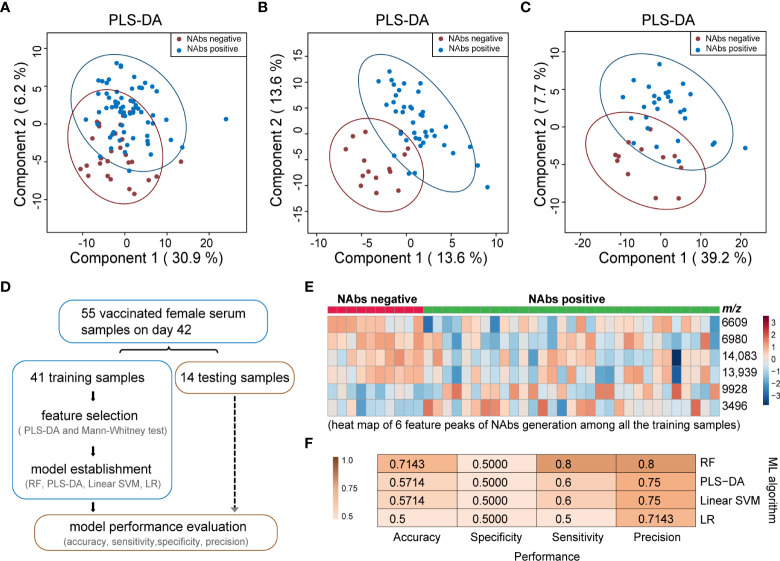
Classification of NAbs positive and negative based on MALDI-TOF MS serum peptidome. **(A)** PLS-DA analysis of all the 95 samples collected on day 42 to classify NAbs positive and negative; **(B)** PLS-DA analysis of 55 samples collected on day 42 from female individuals to classify NAbs positive and negative; **(C)** PLS-DA analysis of 40 samples collected on day 42 from male individuals to classify NAbs positive and negative; **(D)** a general scheme for feature selection and model establishment to classify NAbs positive and negative; **(E)** heat map of six feature peaks of NAbs generation among all the training samples; **(F)** performance of four classification models on the 14 test samples for NAbs generation prediction. N: negative, P: positive, RF: random forest, PLS-DA: partial least squares discriminant analysis, SVM: linear support vector machine, LR: logistic regression. The colored ovals represent 95% confidence interval.

Serum samples from day 42 with the NAbs positive rate of 73% were selected to study the correlation between MALDI-TOF serum peptidome profile and NAbs generation. All the samples were divided into two cohorts, NAbs positive and NAbs negative on day 42. As shown in **Figure 5A**, PLS-DA analysis of the MALDI-TOF mass spectra cannot well differentiate the two cohorts. Then, gender-specific PLS-DA analyses were conducted. When the 55 female samples were analyzed, the classification performance was significantly enhanced, as shown in **Figure 5B**. The overlap of the 95% confidence interval of the NAbs positive and NAbs negative included only 6 positive and 2 negative samples. When the 40 male samples were analyzed, the overlap of the 95% confidence interval of the NAbs positive and NAbs negative included 7 positives and 6 negatives samples, as shown in **Figure 5C**. Better classification performance could be achieved with the gender-specific analyses, and the classification performance was better for female than male, indicating that gender can be a significant factor influencing the serum peptidome related to NAbs generation.

The samples collected on day 42 from female participants were then selected to explore the feasibility of building a classification model to identify NAbs generation based on MALDI-TOF serum peptidome. **Figure 5D** summarized the steps of feature selection and model establishment. All the 55 samples from female participants were divided into a training set (41 samples, 10 negative and 31 positive) and a test set (14 samples, 4 negative and 10 positive). Six significant feature peaks were obtained from the training set, including m/z 3496, m/z 6609, m/z 6980, m/z 9928, m/z 13,939 and m/z 14,083 (**Figure S9**; **Table S5**). As the heatmap represented, 4 features (m/z 6609, m/z 6980, m/z 13939 and m/z 14,083) were more abundant in the females with NAbs negative, and 2 features (m/z 3496 and m/z 9928) were more abundant in the females with NAbs positive (**Figure 5E**). With the 6 features, we tried to build a classification model to distinguish females with and without NAbs production. Verification was carried out on the 14 unlabeled test samples (**Table S6**). Four machine learning methods consisting of random forest (RF), PLS-DA, linear support vector machine (SVM) and logistic regression (LR) were employed to build the models, and compared in the aspects of accuracy, sensitivity, specificity and precision. The results showed that all algorithms had a high precision of over 70% but a relatively low specificity of 50% (**Figure 5F**). The accuracy, precision and sensitivity of the RF-based model all outnumbered 70%, better than LR, linear SVM and PLS-DA-based models (**Figure 5F**). By proteomic analysis, four feature peaks were identified as proteins or protein fragments, including integral membrane protein 2B (m/z 6980), complement component C1q receptor (m/z 6609), platelet factor 4 (PF4) (m/z 9928) and MBL (m/z 14,083) (**Table S7**).

## Discussion

In this study, we profiled the serum peptidome changes induced by CoronaVac vaccination by MALDI-TOF MS, and correlated the significant changes in serum peptidome with the immune protection effect. The significant figures related to vaccination were identified as proteins or protein fragments closely associated with the pathways of oxygen transport, neutrophil degranulation, complement system, and hemostasis. We have also profiled the TAbs and NAbs generation, as well as serum cytokines and lymphocytes subpopulations. The regulation of serum cytokines and lymphocytes subpopulations also suggested neutrophil degranulation.

Modulation of CD3+CD8+ T cells detected in our study provides evidence of stimulated neutrophil under the influence of CoronaVac. Different evidence provided by proteomic, hematological and inflammatory analyses supported a significant increase of neutrophils in severe COVID-19 patients (31), and high level of neutrophils were found in the bronchoalveolar lavage of COVID-19 patients (32–34). A clinical study on COVID-19 hospitalized patients emphasized that the development and activation of neutrophil is a prominent signature of critically ill patients (35). IL-8 is a key chemokine recruiting neutrophils (36). It was reported that IL-8 can act as a marker of COVID-19 severity from non-hospitalized, non-ICU to ICU patients (35). A study confirmed the high IL-8 levels and increased number of neutrophils comparing COVID-19 patients with healthy controls (37). Yadav et el. reported that IL-8 and RBD IgG elevated in rhesus macaques with the administration of inactivated SARS-CoV-2 vaccine BBV152 (36). We also observed the significant upregulation of IL-8 in human serum after CoronaVac vaccination. Similar to COVID-19 infection, neutrophil degranulation was probably activated by the administration of CoronaVac.

Platelet basic protein, CD59 glycoprotein, LRG1 and complement component C1q receptor are proteins associated with neutrophil degranulation, and are significantly correlated with CoronaVac injection. Platelet basic protein is a platelet-derived growth factor belonging to the CXC chemokine family. It has been reported that the protein can down-regulate the function of neutrophil, and can desensitize platelet-derived neutrophil-activating peptide 2–induced neutrophil degranulation (38). In our study, we observed the significant downregulation of platelet basic protein, which can benefit the activation of neutrophil. CD 59 glycoprotein can protect host cells from lysis by complement system attack and is involved in lymphocyte signal transduction. A study of transcriptional profiling indicated that the complement gene of CD59 glycoprotein down-regulated in COVID-19 patients, compared with negative controls (39). Here, we observed downregulation of CD59 glycoprotein after injection of the inactivated SARS-CoV-2 vaccine. LRG1 is a member of the leucine-rich repeat (LRR) family proteins. A proteomic study pointed out that LRG1 upregulated in severe COVID-19 patients as a function of IL-6 levels (40). This is opposite to the regulation direction in CoronaVac vaccinated people found in our study. Also, the changes of IL-6 in serum after vaccination were not significant in our study. Complement component C1q receptor, which is a critical protein belonging to the complement system, upregulated in human blood after receiving the inactivated virus vaccine. The protein can enhance antibody neutralization activity by binding to antibodies (41–43). It is released from its synthetic place in liver to bloodstream, and the process can be accelerated by virus infection (44). A clinical study focusing on the immunology of 71 confirmed COVID-19 cases found that complement component C1q dropped in severe COVID-19 patients significantly compared with mild-ill patients (31). The CoronaVac-induced regulation of complement C1q subcomponent was also detected by Jiang et al (19).

A recent study profiled the changes of the complement system signaling and associated inflammatory mediators between COVID-19 patients and healthy controls (37). The complement system is part of the immune system that enhances the clearance of antibodies and phagocytes, promotes inflammation, and attacks the cell membranes of pathogens. Complement system is a component of native plasma that plays key roles in human immunity, especially in innate immune response (42, 45), and can neutralize enveloped or non-enveloped viruses in case of virus infection (42). Following infection, macrophages are induced by the complement system anaphylatoxins to generate pro-inflammatory cytokines and chemokines. Our study showed that the complement system can be activated by CoronaVac vaccination. There are three complement system activation pathways, including the classical, the alternative and the MBL pathways (42, 46). The upregulation of complement component C1q receptor demonstrated the activation of the classical pathway of the complement system. We found that the level of MBL protein descended after CoronaVac vaccination. MBL is a pattern recognition molecule of the innate immune system. Jiang et al. detected the changes of mannan-binding lectin serine proteases induced by CoronaVac (19). The reduction of MBL serum level or MBL protein concentration has been observed in SARS patients compared to healthy people (45, 47). There were studies reporting that MBL can activate the complement system by binding to SARS-CoV and SARS-CoV-2 spike protein (45, 48, 49). Yu et al. pointed out that recombinant SARS-CoV-2 proteins can activate MBL and hence the MBL pathways for the complement system activation (50). Based on the previous reports, we speculate that inactivated coronavirus in the vaccine may retain the activity of binding MBL, and the complement system can also be activated by vaccination via the MBL pathway.

It is worth noting that the subunits of Hb alpha and Hb beta were significantly downregulated in the CoronaVac vaccinated people. Hb is mainly made up of alpha and beta subunits and iron-containing heme groups. The plummet of hemoglobin subunits prompted that Hb in blood descended after vaccination with the inactivated virus. Previous studies have shown that Hb decreased in COVID-19 infected patients, especially in severely ill patients (51). Wenping Zhang et al. proposed Hb as one of the diagnosis indicators to predict the severity of COVID-19 patients (52). The decline of hemoglobin concentration may correspond to an aggravated clinical condition of patients (51, 53, 54). In the early stage of COVID-19 outbreak, a hypothesis that COVID-19 virus may bind to hemoglobin, release iron ions from porphyrin and damage the oxygen binding ability of hemoglobin was proposed (55). The hypothesis attracted many researchers to focus on studying the interaction between Hb and coronavirus (38, 53, 56). However, no study can reveal the binding mechanism between Hb and coronavirus to date. The change of Hb after vaccination deserves more attention, and long-term surveillance of COVID-19 vaccine safety is necessary, especially on people with hemoglobin-related diseases.

Our study also revealed the downregulation of CD99 antigen. CD99 is involved in T-cell adhesion processes. In 2021, Siwy et al. demonstrated highly significant reduction of CD99 in severe/critical COVID-19, indicating the reduction of endothelial integrity and interference with transendothelial migration of monocytes, neutrophils, and T-cell recruitment (57). The reduction of CD99 in inactivated virus vaccinated people should be further studied.

This study profiled serum cytokines and lymphocyte subpopulation between day 0 and day 28, i.e., before vaccination and one week after complete vaccination. Significant variation of a number of cytokines and lymphocyte subpopulations was observed, suggesting a potential role of helper/inducer T lymphocytes and suppressor/cytotoxic T lymphocytes during the CoronaVac-induced immunization. The regulation was more significant in the relatively young participants (< 50 years) than the middle-aged participants (50-59 years). Since the cytokines were measured from serum without the knowledge of production cells and were only measured on two days, further information on the role of the cytokines in CoronaVac vaccination cannot be obtained in this study. Zhao et al. (18) and Jiang et al. (19) have studied the regulation of cytokines and lymphocytes induced by CoronaVac in a comprehensive way in their respective studies. Jiang et al. characterized the lymphocyte subpopulations before and after vaccination, and found that after the second vaccination, CD8+ cytotoxic T cell levels decreased and the serum NAbs titers increased (19), in consistent with our results, which can suggest a balance between cellular and humoral immune responses dominating at early and late stages of vaccination, respectively.

The analysis of antibody among different age groups revealed that the middle-aged people, especially for people older than 50, displayed the lowest positive rate of NAbs after completion of the CoronaVac vaccination. Indeed, the World Health Organization’s Emergency Use Listing (EUL) authorized the use of CoronaVac in June 2021 with the finding that there is a gap in evidence of the CoronaVac effectiveness for adults over 60 years. An early to mid-stage trial conducted by Sinovac suggested that the immune response of elderly subjects was slightly lower than that of young people. A case-control study observed that the vaccine efficiency descended with increasing age among the elderly people (≥70 years) in Brazil(2021). Recently, a significant reduction in T-cell and antibody responses to inactivated coronavirus vaccination has been reported in people aged 55 years or older (58). With much attention that has been paid to the immune response of elderly people (≥60 years) after vaccination, the protection effectiveness of CoronaVac against SARS-CoV-2 in middle-aged people (50-59 years) deserves more attention.

We tried to distinguish NAbs generation based on serum peptides mass fingerprinting. However, the differentiation performance was not efficient for all samples. Interestingly, gender specific models performed better for the differentiation of NAbs positive and negative individuals, especially on samples from female, demonstrating that gender is a significant factor influencing the serum peptidome correlation to NAbs generation. Comorbidities, such as obesity, have been reported to lower immune response (57). There were only 2 participants with body mass index (BMI) > 30 kg/m2 out of the 95 subjects in our study (**Table S1**). Therefore, obesity factors were not analyzed in association with vaccine immune response.

According to the results of feature annotation by proteomic analysis, NAbs generation is associated with integral membrane protein 2B, complement component C1q receptor, platelet factor 4 (PF4) and MBL. The correlation between complement component C1q and MBL with the immunogenicity via the activation of the complement system has been discussed above. For integral membrane protein 2B, a recent serological study demonstrated that the antibodies produced by COVID-19 patients showed an unexpected immune response against integral membrane proteins, which can be used to detect SARS-CoV-2 variants or test vaccine effectiveness (59). We found that integral membrane protein 2B was less abundant in the NAbs positive individuals. There have been studies confirming the relationship between NAbs generation induced by COVID-19 vaccination and PF4, and we observed more abundant PF4 in the NAbs positive individuals. Platelets are primarily associated with thrombosis (60). Thrombo-inflammation can be induced by platelet activation, and PF4 levels upregulate significantly in COVID-19 patients and especially critically ill patients (52, 60–63). Positive PF4 can also be induced by adenoviral vector COVID-19 vaccine or RNA COVID-19 vaccine with low clinical relevance. Vaccine-induced immune thrombotic thrombocytopenia (VITT) seldomly occurs after vaccination (64). However, a recent study reported the formation of antigenic complexes between vaccine components and PF4 on the platelet surface driven by electric charge after receiving ChAdOx1 nCoV-19 (AstraZeneca) vaccine, which eventually lead to VITT (65). Whether there is the generation of these similar complexes after receiving CoronaVac vaccine remains to be studied.

There are also limitations of the current study. We performed an observational study to characterize the changes of human serum peptidome after receiving deactivated virus COVID-19 vaccine. The recruited participants were mainly young and middle-aged adults. Only limited information on cytokines and lymphocytes was obtained. We detected the generation of NAbs by immunoassay to assess the immune protection ability induced by CoronaVac. Live virus assays, such as PRNT, is the gold standard to evaluate immune protection, but are restricted by biosafety. False positive antibody test results could be obtained by the immunoassay-based methods due to the insufficient specificity of assay kits. On the other hand, false negative results may also be obtained due to improper specimen handling which can lead to low concentration of antibodies extracted. Nevertheless, in most cases, the measurement of NAbs is still considered as an effective mean of immune protection assessment. The performance of the established model for NAbs generation prediction based on serum peptidome is still unsatisfactory, and the analysis of patient demographic characteristics is not sufficiently in-depth. In the future, it is important to carry out validation work on the NAbs generation assessment methods with large sample sizes and multicenter clinical trials. Long-term serum sampling after COVID-19 vaccination should also be performed to enable an in-depth mapping of the serum peptidome dynamic response to COVID-19 vaccination.

In summary, the method developed in this work can monitor the serum peptidome changes induced by CoronaVac injection and can identify features associated with vaccination and NAbs generation. Similar study can also be applied to other COVID-19 vaccines or vaccines for other infectious diseases. With the method, immune responses induced by vaccination can be conveniently monitored. It is also possible to assess vaccine safety by the method. Pre-marketing studies cannot fully guarantee the safety of a vaccine, and follow-up studies should be conducted to re-evaluate the efficacy and safety of vaccines after the product being licensed. The new method developed in our study has the advantage of high throughput, low cost and easy-operation, thereby is especially suitable for large-scale post-marketing monitoring of the efficacy and safety of developed vaccines

## Data availability statement

The mass spectrometry proteomics data have been deposited to the ProteomeXchange Consortium (http://proteomecentral.proteomexchange.org) via the iProX partner repository (66) with the dataset identifier PXD036159.

## Ethics statement

The studies involving human participants were reviewed and approved by Ethical Committee of Chongqing General Hospital. Written informed consent for participation was not required for this study in accordance with the national legislation and the institutional requirements.

## Author contributions

WZ performed the vaccination and collected the sample. DL analyzed the data and wrote the draft of the manuscript. BX collected the mass spectrometry data. LX assisted in the sample collection. QL assisted in the mass spectrometry analysis. XL assisted in the data analysis. ZL assisted in the sample collection. JZ assisted in the data analysis. WS was involved in the study design. QM designed the study and supervised the mass spectrometry analysis. LQ designed the study, supervised the data analysis, and finalized the manuscript. PL designed the study and supervised the clinical model establishment. All authors contributed to the article and approved the submitted version.

## Funding

This work was supported by the Ministry of Science and Technology of China (MOST, 2020YFF0426500), the Chongqing Department of Science and Technology: 2021 Chongqing Talent Program (2021-07-12-230) and the National Natural Science Foundation of China (NSFC, 22022401, 22074022, 21934001).

## Conflict of interest

Authors BX, QL and QM were employed by Bioyong Technologics, Inc.

The remaining authors declare that the research was conducted in the absence of any commercial or financial relationships that could be construed as a potential conflict of interest.

## Publisher's note

All claims expressed in this article are solely those of the authors and do not necessarily represent those of their affiliated organizations, or those of the publisher, the editors and the reviewers. Any product that may be evaluated in this article, or claim that may be made by its manufacturer, is not guaranteed or endorsed by the publisher.
