# Supplementary material for: Serum peptidome profiles immune response of COVID-19 Vaccine administration
Source: Front Immunol. 2022 Aug 24;13:956369. doi: 10.3389/fimmu.2022.956369 (PMC9450691; doi:10.3389/fimmu.2022.956369)
Supplement: Supplementary file 1 [file DataSheet_1.pdf]

Supplementary Material

1 Supplementary Data

Supplementary Dataset 1: The peak intensity matrix of all the samples.

2 Supplementary Figures and Tables

2.1 Supplementary Figures

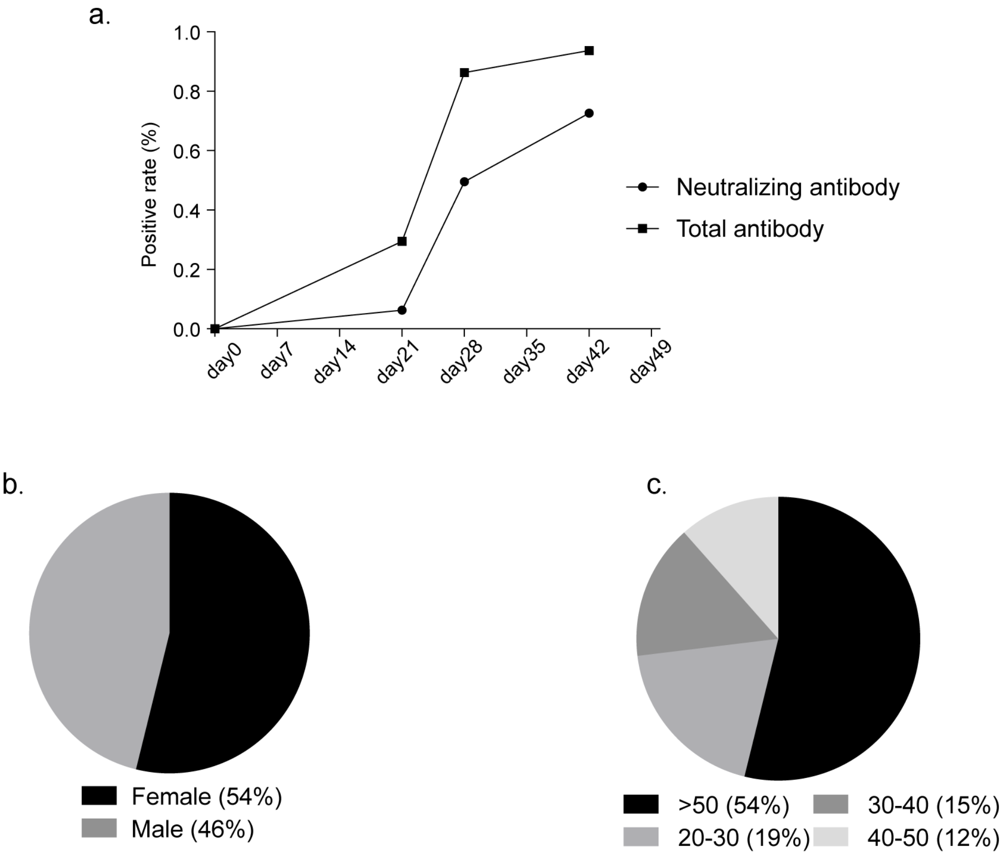

Fig. S1. a) Antibody analysis of the 95 participants before and after vaccination, b) sex distribution of the 26 people with Nabs negative on day 42, c) age distribution of the 26 people with NAbs negative on day 42.

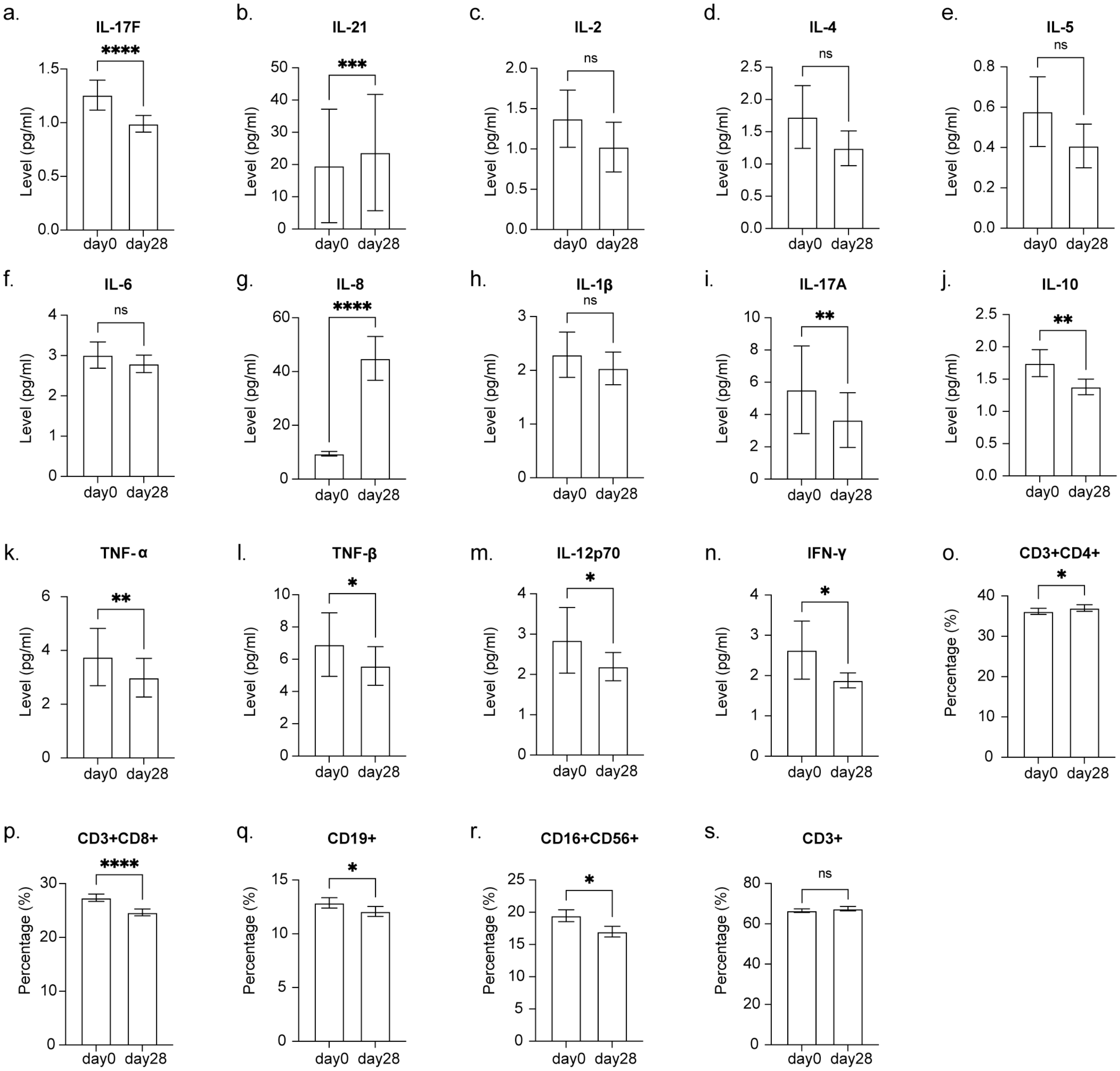

Fig. S2. Comparison of cytokines and lymphocytes subpopulations between the samples collected on day 0 and day 28 for all the participants. Error bars show standard error of mean among samples. P-value was calculated by the Wilcoxon test. Asterisks represent statistically significant difference between groups (*: p-value < 0.05, **: p-value < 0.01, ***: p-value < 0.001, ****: p-value < 0.0001).

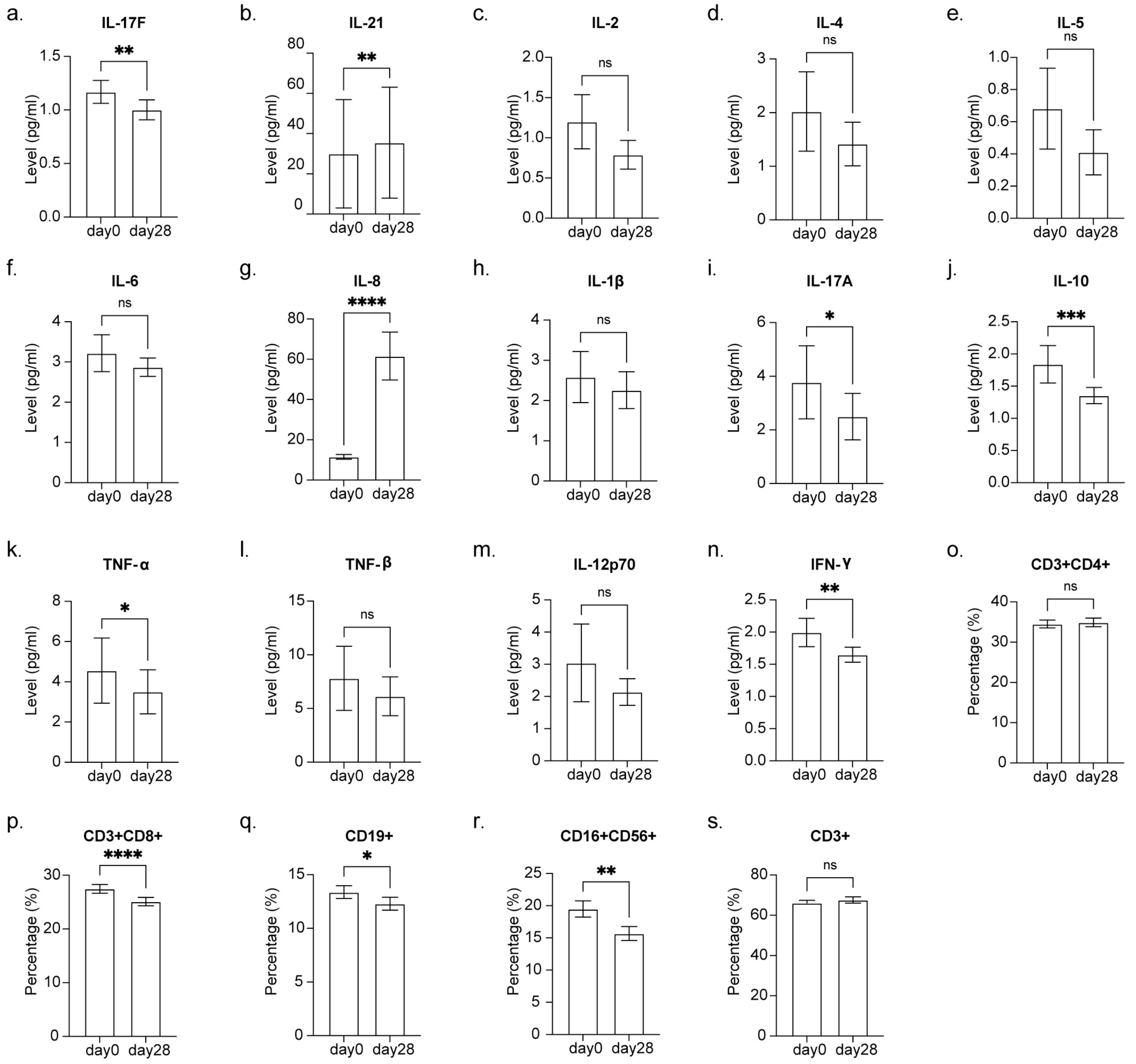

Fig. S3. Comparison of cytokines and lymphocytes subpopulations between the samples collected on day 0 and day 28 for all the participants with age < 50. Error bars show standard error of mean among samples. P-value was calculated by the Wilcoxon test. Asterisks represent statistically significant difference between groups (*: p-value < 0.05, **: p-value < 0.01, ***: p-value < 0.001, ****: p-value < 0.0001).

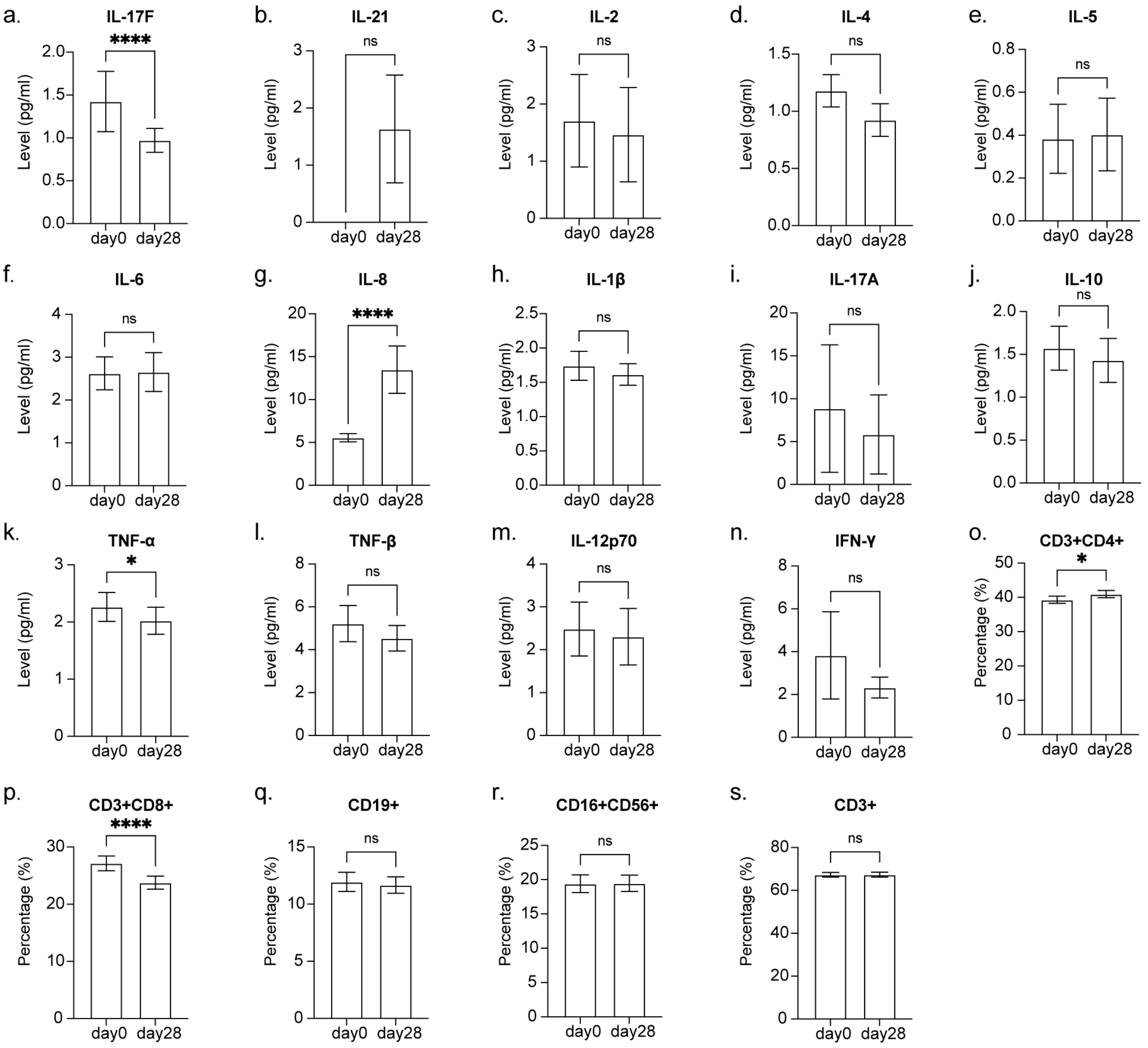

Fig. S4. Comparison of cytokines and lymphocytes subpopulations between the samples collected on day 0 and day 28 for all the participants with age > 50. Error bars show standard error of mean among samples. P-value was calculated by the Wilcoxon test. Asterisks represent statistically significant difference between groups (*: p-value < 0.05, ****: p-value < 0.0001).

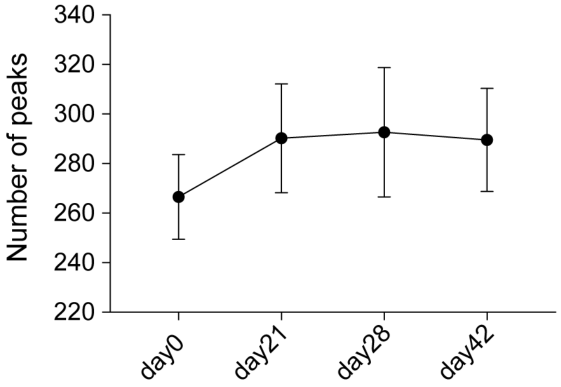

Fig. S5. Number of peaks on MALDI-TOF mass spectra of serum peptidome from 95 participants on day 0, day 21, day 28 and day 42. Error bars show standard deviation among samples.

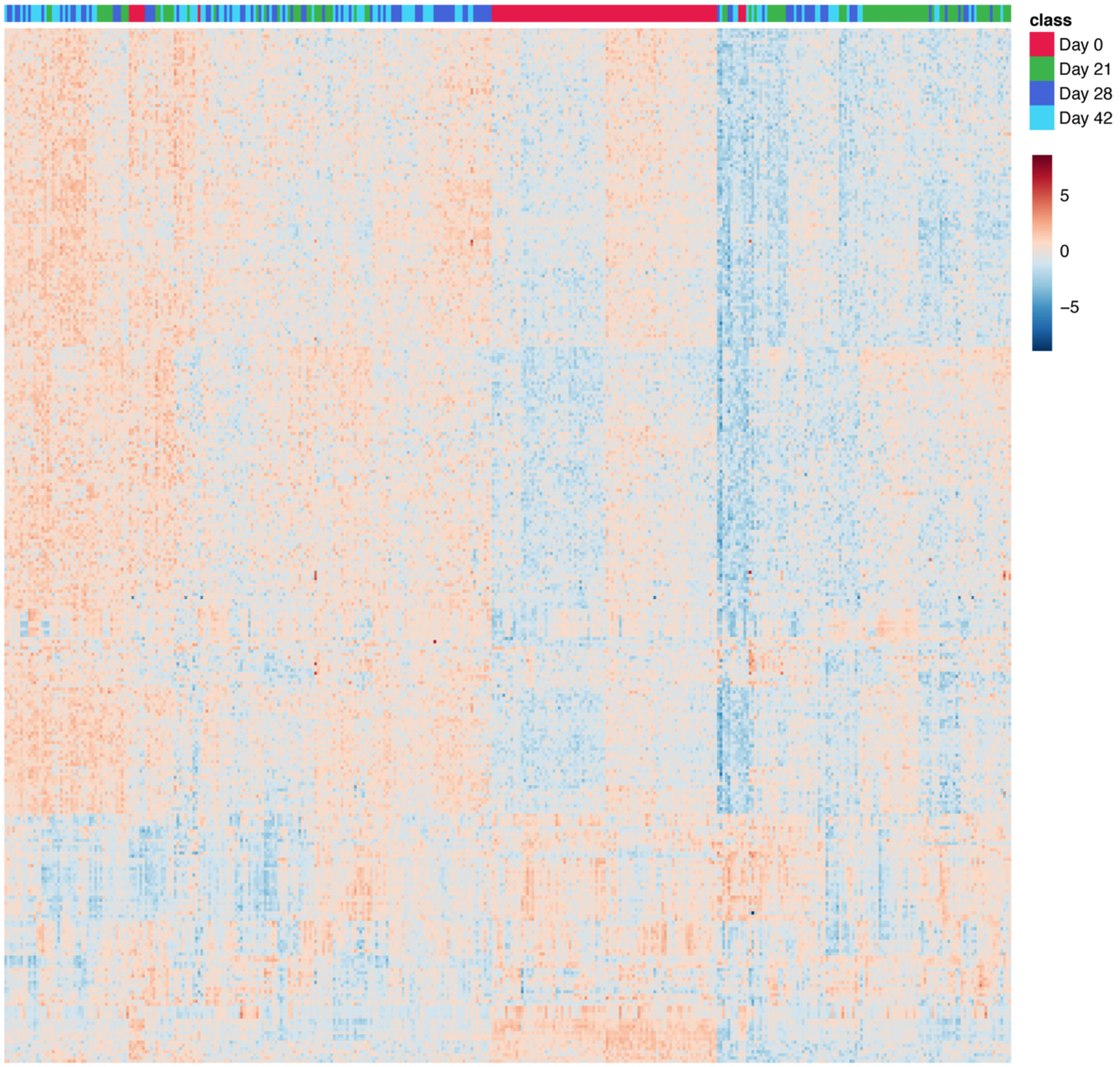

Fig. S6. Heat map based on cluster analysis of MALDI-TOF MS features collected at four time points for all participants before and after vaccination. Distance measure using Euclidean, and clustering algorithm using ward.D.

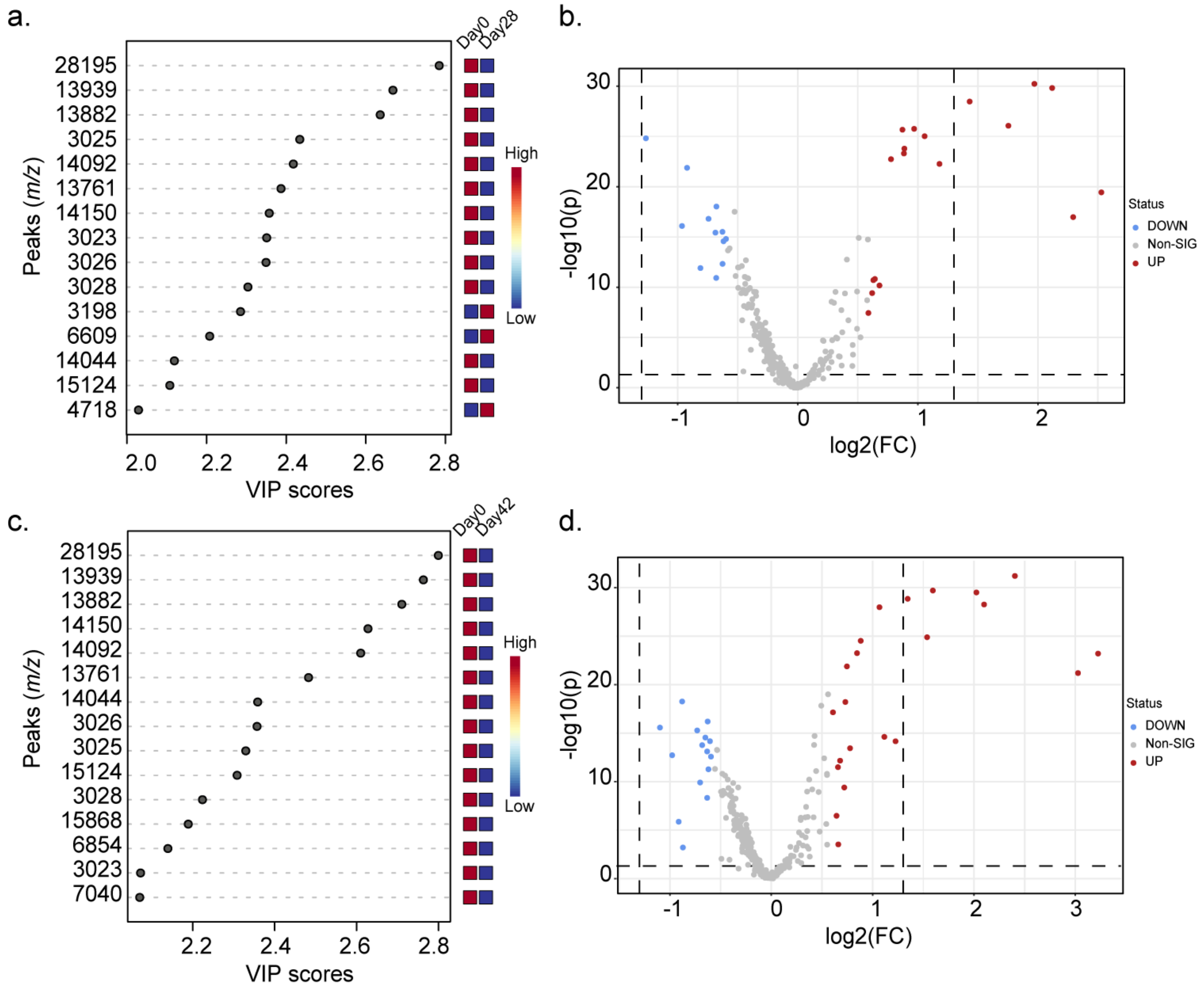

Fig. S7. a) Top 15 features by PLS-DA between the MALDI-TOF MS features of samples collected on day 0 and day 28 ranked by the decrease in VIP values; b) volcano-plot between MALDI-TOF MS features of samples collected on day 0 and day 28 (day 0/day 28); c) Top 15 features by PLS-DA between the MALDI TOF MS features of samples collected on day 0 and day 42 ranked by the decrease in VIP values; d) volcano-plot between MALDI TOF MS features of samples collected on day 0 and day 42 (day 0/day 42). The colored dots represent significant features by volcano-plot (fold change (FC) > 1.5, and p-value < 0.05). P-value was calculated by the Wilcoxon test.

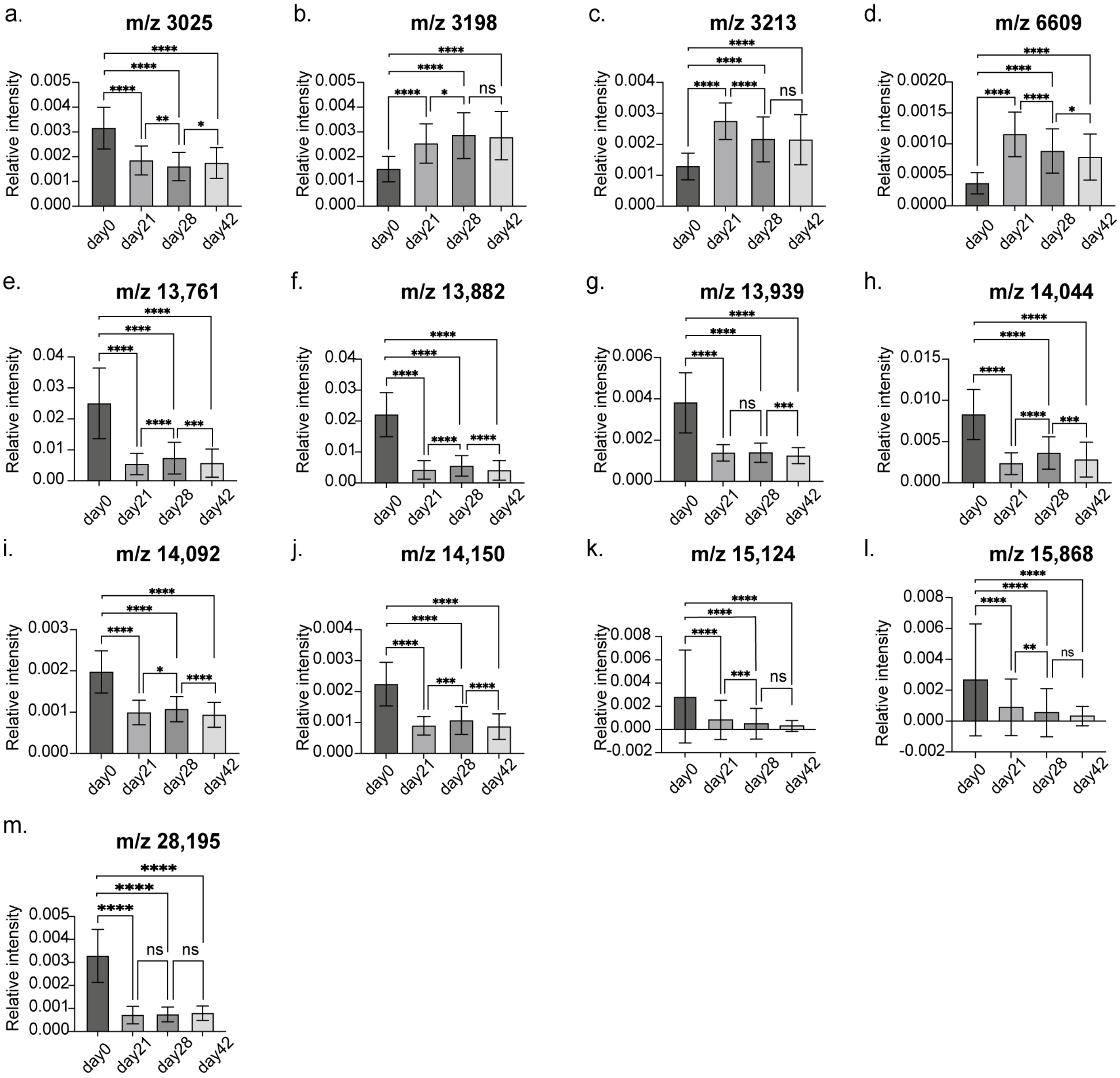

Fig. S8. The relative intensity of the 13 feature peaks before vaccination and during the 6-week recovery phase after vaccination. Error bars show standard deviation of mean among samples. P-value was calculated by the Wilcoxon test. Asterisks represent statistically significant differences between groups (*: p-value < 0.05, **: p-value < 0.01, ***: p-value < 0.001, ****: p-value < 0.0001).

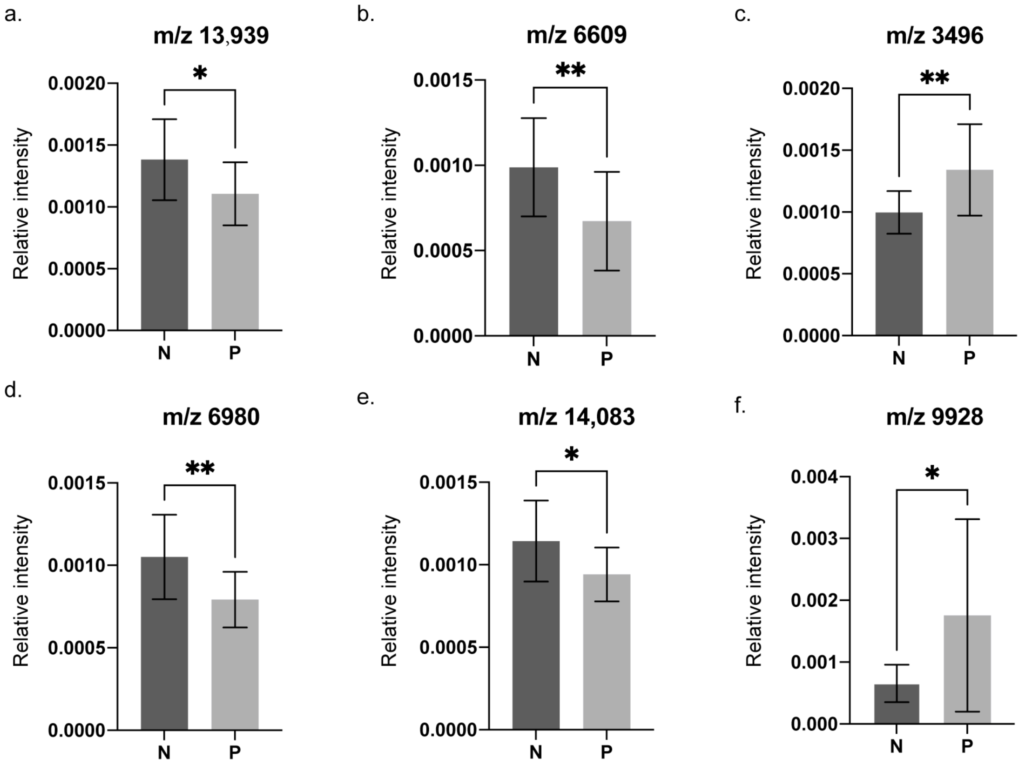

Fig. S9. Relative intensity of 6 feature peaks of NAbs generation for samples collected on day 42 from female participants. Error bars show standard deviation of mean among samples. P-value was calculated by the Mann-Whitney test. Asterisks represent statistically significant differences between groups (*p-value < 0.05, **p-value < 0.01). N: NAbs negative; P: NAbs positive.

2.2 Supplementary Tables

Table S1. Demographics of participants

**Sex=no.**					
Male (%)			40 (42.1)		
Female (%)			55 (57.9)		
**Age-year**					
Range	20-29	30-39	40-49	50-54	55-59
People count (%)	28(29.4)	16(16.8)	18(18.9)	11(11.6)	22(23.2)
Mean ± SD	25.0±2.5	34.3±3.5	44.7±2.9	51.6±0.9	56.6±1.3
Median (IQR)	25.0±4.0	35.0±7.3	45.0±4.8	52.0±1.0	57.0±2.0
NAbs production on day 42 (%)	53.6	62.50	55.60	27.3	40.9
**BMI**					
Value (kg/m^2)	Below 18.5	18.5-24.9	25-29.9	Above 30	
Range	Underweight	Normal weight	Pre-obesity	Obesity	
People count (%)	4 (4.2)	60 (63.2)	29 (30.5)	2 (2.1)	
Mean ± SD	18.1±0.3	22.2±1.4	26.8±1.0	31.8±0.8	
Median (IQR)	18.2 (0.4)	22.4 (2.1)	26.7 (1.1)	31.8 (0.8)	
**Antibody**					
Sampling time	Day 0	Day 21	Day 28	Day 42	
Total antibody (%)	0 (0.0)	28 (29.5)	82 (86.3)	90 (94.7)	
NAbs (%)	0 (0.0)	6 (0.1)	47 (49.5)	69 (72.6)	

BMI: body mass index. The recommended levels are adapted from the global WHO recommendation of over 30 as an obese BMI. SD: standard deviation. IQR: interquartile range of feature intensity.

Table S2. Intensities of feature peaks of vaccination (Day 0 and Day 28)

m/z	Day 0		Day 28		Raw p-value**	Adjusted p-value***	VIP score
	Median (IQR)* (E-03)	Q_1–Q_3* (E-03)	Median (IQR)* (E-03)	Q_1–Q_3* (E-03)			
3025	3.08 (0.9)	2.64-3.54	1.55 (0.61)	1.26-1.87	1.2983E-34	4.2195E-34	2.351
3198	1.49 (0.71)	1.15-1.86	2.91 (1.15)	2.27-3.42	5.0417E-29	8.1928E-29	2.2853
6609	0.385 (0.223)	0.248-0.471	0.860 (0.418)	0.652-1.07	1.651E-26	2.3847E-26	2.2079
15124	1.46 (2.854)	0.476-3.33	0.154 (0.248)	0.103-0.351	1.3888E-23	1.6413E-23	2.1078
13882	21.5 (10.1)	17.1-27.2	5.13 (3.99)	3.46-7.45	1.9766E-44	8.5651E-44	2.6362
13761	23.7 (13.3)	17.4-30.7	6.81 (4.94)	4.19-9.13	1.009E-32	2.1861E-32	2.387
13939	3.67 (1.95)	2.75-4.70	1.35 (0.41)	1.19-1.60	2.6798E-46	1.7419E-45	2.6681
14092	1.97 (0.67)	1.68-2.35	1.08 (0.348)	0.902-1.25	5.9111E-34	1.5369E-33	2.418
28195	3.30 (1.62)	2.49-4.11	0.758 (0.436)	0.556-0.992	3.4619E-54	4.5005E-53	2.7843
14150	2.14 (0.92)	1.71-2.63	1.05 (0.64)	0.71-1.35	1.3458E-31	2.4993E-31	2.3575
14044	8.02 (3.6)	6.50-10.1	3.60 (2.71)	2.41-5.12	6.6495E-24	8.6443E-24	2.1192
3213	1.25 (0.541)	0.989-1.53	2.20 (0.92)	1.74-2.66	2.1796E-21	2.3613E-21	2.0244

*: IQR: interquartile range of feature intensity; Q_1: the lower quartile value; Q_3: the upper quartile value.
**: raw p-value was calculated by the Wilcoxon test.
***: Adjusted p-value: raw p-value was adjusted for multiple hypothesis testing using a Benjamini-Hochberg correction.

Table S3. Intensities of feature peaks of vaccination (Day 0 and Day 42)

m/z	Day 0		Day 42		Raw p-value**	Adjusted p-value***	VIP score
	Median (IQR)* (E-03)	Q_1–Q_3* (E-03)	Median (IQR)* (E-03)	Q_1–Q_3* (E-03)			
3025	3.08 (0.9)	2.64-3.54	1.67 (0.62)	1.44-2.06	2.1942E-30	3.8399E-30	2.3295
3198	1.49 (0.71)	1.15-1.86	2.64 (1.52)	2.08-3.60	6.2067E-22	7.8994E-22	2.0498
15124	1.46 (2.854)	0.476-3.33	0.137 (0.107)	0.103-0.210	1.1972E-29	1.8622E-29	2.3083
15868	1.22 (3.278)	0.372-3.65	0.106 (0.102)	0.071-0.173	8.5715E-26	1.2E-25	2.189
13882	21.5 (10.1)	17.1-27.2	3.46 (4.35)	1.51-5.86	1.3764E-48	6.4234E-48	2.7104
13761	23.7 (13.3)	17.4-30.7	4.93 (5.83)	2.29-8.12	1.823E-36	4.2536E-36	2.4826
13939	3.67 (1.95)	2.75-4.70	1.25 (0.45)	1.02-1.47	3.5711E-52	2.4998E-51	2.763
14092	1.97 (0.67)	1.68-2.35	0.934 (0.336)	0.754-1.09	1.1375E-42	3.1851E-42	2.61
28195	3.30 (1.62)	2.49-4.11	0.789 (0.371)	0.625-0.996	6.4943E-55	9.0921E-54	2.7995
14150	2.14 (0.92)	1.71-2.63	0.82 (0.501)	0.619-1.12	1.2199E-43	4.2696E-43	2.6278
14044	8.02 (3.6)	6.50-10.1	2.85 (2.69)	1.20-3.89	1.8906E-31	3.7812E-31	2.3586

*: IQR: interquartile range of feature intensity; Q_1: the lower quartile value; Q_3: the upper quartile value.
**: raw p-value was calculated by the Wilcoxon test.
***: Adjusted p-value: raw p-value was adjusted for multiple hypothesis testing using a Benjamini-Hochberg correction.

Table S4. Annotation of feature peaks of vaccination.

Matched Features (m/z)	Gene Name	Protein Accession	Protein	Function
3025	CD99	P14209	CD99 antigen (75-105)	ENOG503JHJ5: S, CD99 antigen like protein 2
6609	CD93	Q9NPY3	Complement component C1q receptor (242-303)	ENOG503J31H: T, Signal transduction mechanisms; complement component C1q binding
13,882	PPBP	P02775	Platelet basic protein	ENOG503JI3M: T, Signal transduction mechanisms. CXCR chemokine receptor binding
14,044	HBD	P02042	Hemoglobin subunit delta (19-147)	ENOG503JGFD: C, Energy production and conversion. hemoglobin subunit
14,092	MBL2	P11226	Mannose-binding protein C (100-227)	ENOG503J318: W, Extracellular structures. mannose-binding lectin (protein C) 2
14,150	CD59	P13987	CD59 glycoprotein	ENOG503JHW4: T, Signal transduction mechanisms. CD59 molecule, complement regulatory protein
15,124	HBA1	P69905	Hemoglobin subunit alpha (2-142)	ENOG503JGIH: C, Energy production and conversion. oxygen carrier activity
15,868	HBB	P68871	Hemoglobin subunit beta (2-147)	ENOG503JGFD: C, Energy production and conversion. hemoglobin subunit
28,195	LRG1	P02750	Leucine-rich alpha-2-glycoprotein (94-347)	ENOG503JA7H: T, Signal transduction mechanisms. Leucine rich repeat C-terminal domain

Table S5. Intensities of feature peaks of NAbs generation in the training cohort.

m/z	Positive group		Negative group		Raw p-value**	Adjusted p-value***	VIP score
	Median (IQR)* (E-03)	Q_1–Q_3* (E-03)	Median (IQR)* (E-03)	Q_1–Q_3* (E-03)			
13939	1.11 (0.355)	0.915-1.27	1.35 (0.32)	1.23-1.55	0.014057	0.01968	2.3601
6980	0.764 (0.195)	0.672-0.867	1.04 (0.343)	0.887-1.23	0.004193	0.0081819	2.7228
3496	1.25 (0.42)	1.09-1.51	1.02 (0.315)	0.835-1.15	0.0018686	0.0065401	2.7516
14083	0.954 (0.196)	0.834-1.03	1.14 (0.336)	0.964-1.30	0.028303	0.028303	2.1904
6609	0.722 (0.474)	0.407-0.881	1.09 (0.394)	0. 806-1.20	0.0046754	0.0081819	2.6133
9928	1.17 (1.704)	0.546-2.25	0.644 (0.369)	0.411-0.780	0.028303	0.028303	2.3028

*: IQR: interquartile range of feature intensity; Q_1: the lower quartile value; Q_3: the upper quartile value.
**: raw p-value was calculated by the Mann-Whitney test.
***: Adjusted p-value: raw p-value was adjusted for multiple hypothesis testing using a Benjamini-Hochberg correction.

Table S6. Prediction results of NAbs generation using different machine learning models in the test cohort.

Sample Name	True	RF	PLS-DA	SVM	LR
A4001	N	N	N	N	N
A4006	P	P	N	N	N
A4009	P	P	P	P	P
A4014	N	P	P	P	P
A4032	N	P	P	P	P
A4052	P	P	P	P	P
A4055	N	N	N	N	N
A4070	P	N	N	N	N
A4072	P	P	P	P	P
A4076	P	P	P	P	P
A4078	P	P	N	N	N
A4080	P	N	N	N	N
A4083	P	P	P	P	P
A4097	P	P	P	P	N

N: negative, P: positive, RF: random forest, PLS-DA: partial least squares discriminant analysis, SVM: linear support vector machine, LR: logistic regression.

Table S7. Annotation of the feature peaks of NAbs generation for samples collected on day 42 from female participants.

Matched Features (m/z)	Gene Name	Protein Accession	Protein	Function
6980	ITM2B	Q9Y287	Integral membrane protein 2B	ENOG503J93I: S, negative regulation of amyloid precursor protein biosynthetic process
6609	CD93	Q9NPY3	Complement component C1q receptor	ENOG503J31H: T, Signal transduction mechanisms; complement component C1q binding
9928	PF4	P02776	Platelet factor 4	ENOG503JHWI: T, Signal transduction mechanisms.<br>platelet factor 4
14083	MBL2	P11226	Mannose-binding protein C	ENOG503J318: W, Extracellular structures.<br>mannose-binding lectin (protein C) 2
